# Mapping Concurrent Wasting and Stunting Among Children Under Five in India: A Multilevel Analysis

**DOI:** 10.3389/ijph.2023.1605654

**Published:** 2023-06-07

**Authors:** Bikash Khura, Parimala Mohanty, Aravind P Gandhi, Lipilekha Patnaik, Abhishek Mewara, KeertiBhusan Pradhan, Bijaya Kumar Padhi

**Affiliations:** ^1^ International Institute for Population Sciences, Mumbai, India; ^2^ Department of Community Medicine, Institute of Medical Sciences and SUM Hospital, Siksha 'O' Anusandhan Deemed to Be University, Bhubaneswar, India; ^3^ Department of Community Medicine, ESIC Medical College and Hospital, Hyderabad, India; ^4^ Department of Medical Parasitology, Postgraduate Institute of Medical Education and Research, Chandigarh, India; ^5^ Public Health Management, Chitkara University, Chandigarh, Punjab, India; ^6^ Department of Community Medicine and School of Public Health, Postgraduate Institute of Medical Education and Research, Chandigarh, India

**Keywords:** undernutrition, wasting, stunting, under-five children, India

## Abstract

**Objectives:** The study aims to examine the coexisting forms, patterns, and predictors of concurrent wasting and stunting (WaSt) among children under five in India.

**Methods:** We used data from the National Family Health Survey to understand the trend and association of WaSt among children under five-year-old in India. Univariate analysis and cross-tabulations were performed for WaSt cases. The association was determined using multilevel binary logistic regression and multilevel regression, and the results were provided as adjusted odds ratios (aOR) with 95% confidence intervals at the significance level of *p* < 0.05.

**Results:** The prevalence of WaSt has decreased from 8.7% in 2005–06 to 5.2 percent in 2019–2020. The proportion of WaSt children grew rapidly from 6 to 18 months, peaked at 19 months (8%), then dropped after 24 months. The prevalence of concurrent wasting and stunting is higher among boys compared to girls. Compared to children of different birth orders, those in the higher birth order are 1.2 times more likely to be WaSt cases (aOR = 1.20, 95% CI = 1.09, 1.33). The education of the mother is strongly correlated with WaSt instances, and children of more educated mothers have a 47% lower chance of being WaSt cases (aOR = 0.63, 95% CI = 0.57, 0.71). Children from wealthy families are 52% less likely to be WaSt cases (aOR = 0.48, 95% CI = 0.43, 0.55).

**Conclusion:** This study emphasizes the importance of concurrent wasting and stunting and its relationship with socioeconomic factors among children under five in India.

## Introduction

Undernutrition is a global public health problem that disproportionately affects low- and middle-income countries. It has various aspects that include wasting, stunting, and underweight [1, 2]. These conditions are associated with a variety of negative health outcomes, including increased morbidity and mortality, impaired physical and cognitive development, poor learning capacity, and reduced educational attainment. It has been estimated that 149 million children and 45 million under 5 years of age are stunted and wasted, worldwide [1]. Among them, India, the second most populous country, accounts for 40% of stunted children, translating to 62 million and 60 million underweight children [3].

The disorders associated with growth and nutrition are a major cause of morbidity and mortality in young children [4, 5], accounting for 45% of the total deaths in children under five [6]. In India, the proportion of deaths attributed to malnutrition was higher (68.2%), translating to 706,000 deaths in 2017 [7]. Nutritional disorders have been shown to perpetuate poverty. Undernutrition affects the physical and cognitive functions of children, ultimately resulting in low productivity [8, 9]. This, in turn, affects the country’s economy as population productivity directly impacts the gross domestic product (GDP). It has been estimated that a 1% loss of height in an adult due to stunting in childhood leads to a productivity loss of 2.4% at the individual level [10]. Extrapolating the productivity loss of individuals to the country level, it has been estimated to negatively impact the GDP by 2%–3%. Therefore, undernutrition has individual, social, and economic costs for the present and future [6].

Growth disorders do not always occur in isolated forms. Two or more aspects of undernutrition can occur in the same child during the same period of time. The concept of the simultaneous occurrence of various forms of growth disorders came up after 2014 [11]. Coexisting Forms of Malnutrition (CFM) have been reported to increase mortality and morbidity, compared with the isolated forms [12, 13]. Globally, simultaneous wasting and stunting (WaSt) was reported to be 3.5% among children under five. However, the burden has regional disparity, with Asia (5%) and Africa (2.9%) having a higher prevalence of the problem than European countries [11]. Few studies have reported a prevalence of coexisting WaSt of up to 12% in these regions [2]. McDonald et al. reported a prevalence of wasting, stunting, and underweight of 5.5% in their meta-analysis of 10 cohorts around the world [13]. Khan et al. in their multilevel analysis from India estimated the prevalence of coexisting wasting and underweight to be 8.1%, while coexisting wasting, stunting, and underweight was 6.5% based on the nationwide survey conducted 8 years ago [14]. The gender of the child was found to be a significant predictor [14]. With the most recent data on undernutrition released from the Indian nationwide survey in 2021, among children under five, 35.5% were stunted, 19.3% were wasted, and 32.1% were underweight. Although the proportion of general wasting, underweight, and stunting has positively improved, the status of severe wasting has worsened from 7.5% in 2015–16% to 7.7% in 2019–21 [15]. Therefore, the interaction between these facets of childhood growth disorders and demographic features might have changed, which needs exploration in the Indian context.

Therefore, the present study was conducted to estimate coexisting forms, patterns of undernutrition, and predictors of coexisting Wasting and Stunting (WaSt) among children in India.

## Methods

### Data Source

The study used data from the latest round of the National Family Health Survey (NFHS-5), which was conducted in 2019–2021. The NFHS offers state and union territory-level data on India’s population, health, and nutrition. The NFHS is carried out under the management of the Global Demographic and Health Survey (DHS). It is a cross-sectional survey carried out under the direction of the Ministry of Health and Family Welfare (MoHFW) (ICF, IIPS). The survey uses a two-stage stratified sampling procedure to represent the 36 Indian states and union territories; the methods and techniques for sampling are covered elsewhere. The primary goal of the NFHS is to provide data on indicators such as nutrition, family planning, domestic violence, maternity and child health, fertility, mortality, and women’s empowerment. In this survey, 747,176 reproductive-age women (15–49) were questioned from 636,699 household samples.

This study aims to examine the pattern and determinant of concurrent stunting and wasting in India, and therefore only includes children under 5 years of age. The final analysis was performed on 199,682 children born within the 5 years prior to the survey after removing flagged observations (with atypical measurements and incomplete/missing information).

### Statistical Analysis

#### Case Definition

Concurrent stunting and wasting are cases of interaction between the two conditions and are defined as: WaSt=WHZ <−2.0 and HAZ <−2 .0.

Composite index of WaSt z score: WaStZ = (WHZ + HAZ)/2 if WHZ < 0 and HAZ < 0; = 0 otherwise; the severity of WaSt: Z < −1 “mild”, Z < −2 “moderate,” Z < −3 “severe”

#### The Degree of Overlap Between Stunting, Wasting, and Underweight

A Venn diagram was used to analyze the degree of overlap between stunting, wasting, and underweight among children aged 0–59 months in India.

This analysis aimed to define the magnitude of the set:

Stunted∩Wasted

And:Stunted∩Wasted∩Underweight

Where ∩ denotes intersection. Set A ∩ B determines that all members of A who are also B (or *vice versa*) make up a set A B. The set-theory equivalent of the Boolean AND operator is the intersection.

#### The Severity of Stunting and Wasting in WaSt Cases

The severity of concurrent stunting and wasting was calculated using the composite index defined above [12].

The explanatory factors were classified into three groups:

Age, sex, birth order, size at birth, recent fever, cough, and diarrhea were considered child characteristics. Mother’s level of anemia, antenatal care, delivery place, and education were considered maternal characteristics. Household variables included drinking water sources, toilet facility, household size, frequency of reading newspapers, radio, and television, cooking fuel consumption, wealth index, place of residence, religion, and caste. All the aforementioned factors were selected based on their availability in the NFHS data and their literature-supported connection to child undernutrition [16].

Cross-tabulations were used to examine the factors associated with WaSt cases, and univariate chi-square analysis was fitted with the predictor variables. After examining all the factors, we used the method suggested by Lawless and Singhal [17] to select the best subset variables and applied the Stata command gvselect for variable selection. We applied a multilevel binary logistic regression to establish the association between the outcome and the predictor variables. We fitted four models to demonstrate the association between the explanatory and outcome variables.

Model 0 was fitted to show the variance in the outcome variable clustered at the primary sampling unit (PSU) level. Model I was fitted after adjusting for age and sex variables. Model II was fitted after considering all explanatory variables whose p-values were significant from the chi-square test, and the final model—model III was fitted by considering the subset variable suggested in the method of Lawless and Singhal [18].

AIC was used to test the fitness of the model and comparison. We used odds ratios (OR) and their 95% confidence intervals (CI) to present the results of the regression analysis. All the analysis was done in STATA version 16.0.

## Results

**FIGURE 1:**
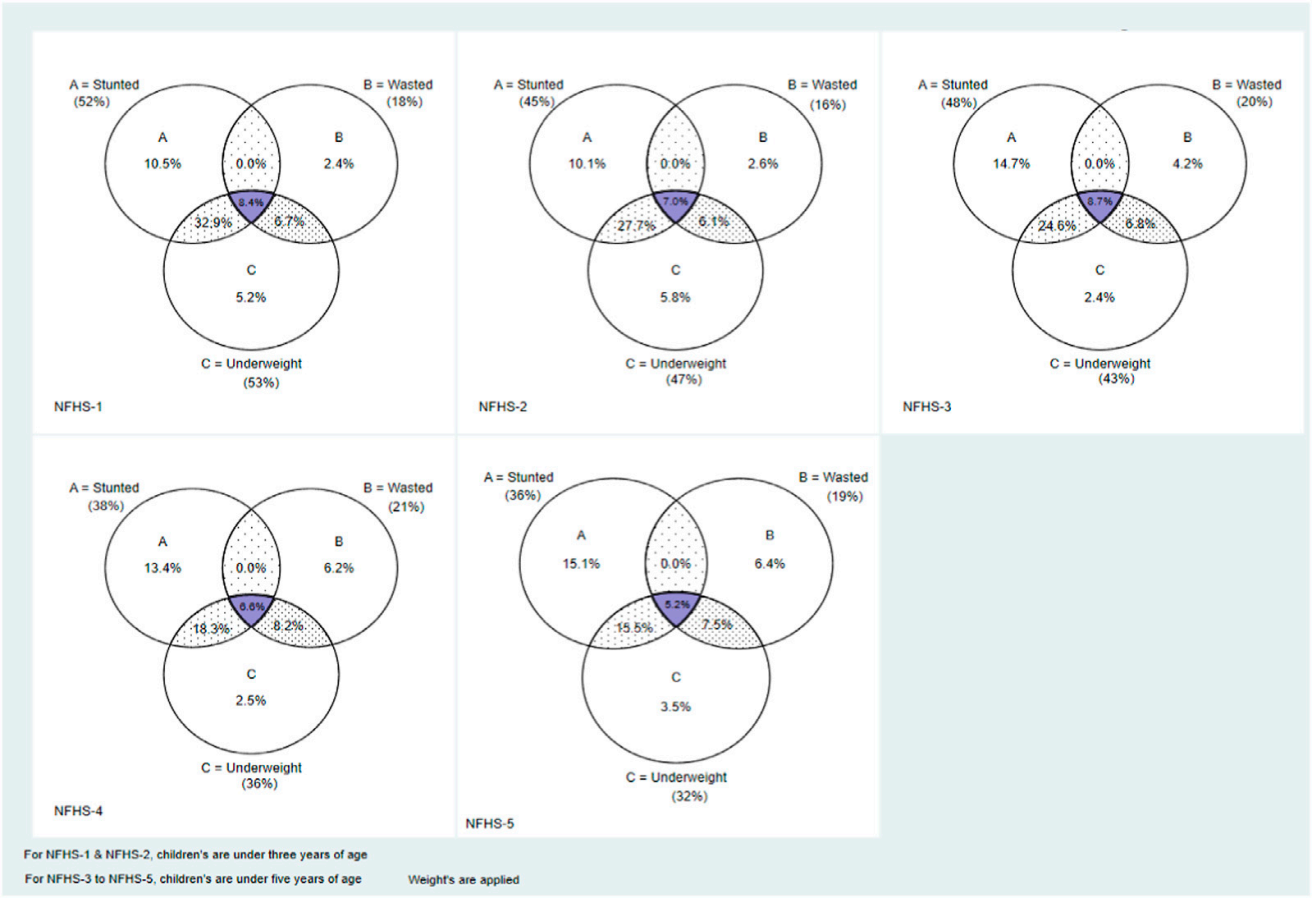
Venn diagram showing the relationship between the set of children who are stunted, wasted, and underweight, India, 1992–2019. For the periods of 1992–93 and 1998–99, the magnitude of the sets was calculated using children from 0 to 35 months. For the periods 2005–2006 to 2019–21, the magnitude of the sets was calculated using children from 0 to 59 months.

Figure 1 shows the relationship between stunting, wasting, and underweight among children under five in India. The results show that children who are simultaneously stunted and wasted are also underweight. This meant that no child in the sample with stunting (WHZ < −2) and wasting (HAZ < −2) had a WAZ 2. The prevalence of WaSt (concurrently wasted and stunted) decreased from 8.7% in 2005–2006 to 5.2% in 2019–21.

### The Age Pattern of WaSt

**FIGURE 2:**
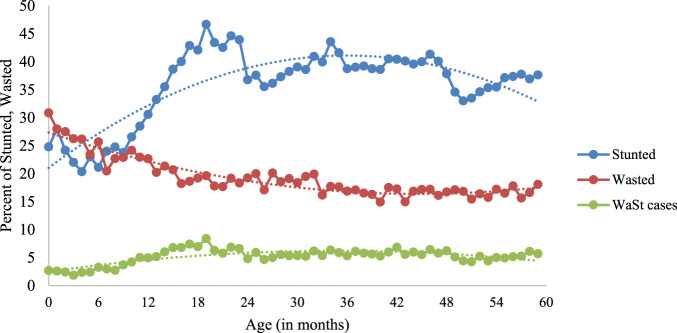
Age pattern of stunting, wasting and WaSt among children 0–59 months of age, India, 2019–21.

Figure 2 shows the age pattern of malnutrition among children under 5 years of age. The proportion of stunting in children increased from 6 to 18 months of age, decreased until the age of 24 months, and increased again until the 36th month. However, in the case of wasting, the prevalence decreased with age. The proportion of children who are simultaneously stunted and wasted expeditiously increased from 6 to 18 months of age, peaked at around 19 months (8%), then began to fall after 24 months. Therefore, age is a key factor in the malnutrition pattern dynamics of these children.

**FIGURE 3:**
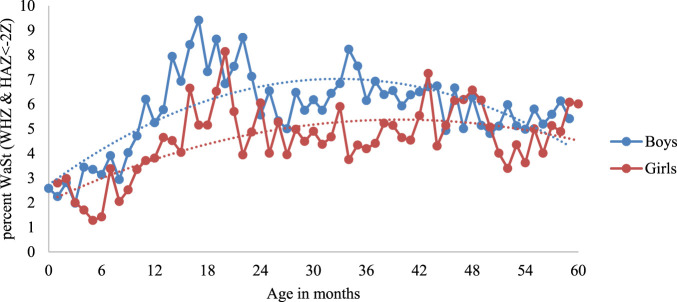
Prevalence of concurrent stunting and wasting by sex of children, India, 2019–21.

Figure 3 further illustrates the sex differences among WaSt cases. The prevalence of concurrent stunting and wasting was higher among boys than among girls. It peaked around the age of 17 months (9%) among boys and 19 months (8%) among girls.

**FIGURE 4:**
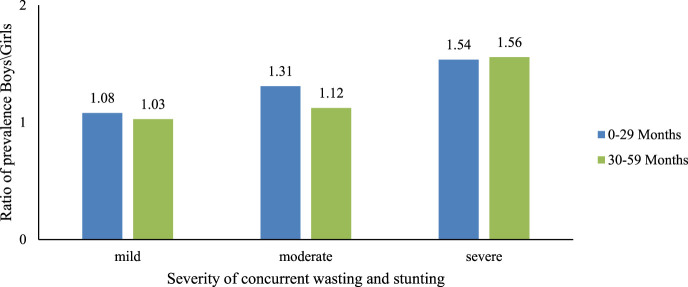
Effect of concurrent severe stunting and wasting on sex differences among children, India, 2019–21.

From the composite index described above, the severity of concurrent stunting and wasting was determined as follows: sum of the z score of height and weight divided by two; if both are negative, else is zero. The severity levels were classified as mild if Z score was less than −1, moderate if the Z score was between −1 and −3 and severe if the Z scores was less than −3. Under 30 months of age, but not beyond, the pattern of sex disparities was proportional to the severity of concurrent WaSt (Figure 4).

Table 1 shows the bivariate analysis of WaSt among children under 5 years of age in India. Children in the 12–23-month age range were observed to have the highest prevalence (6.48%) of WaSt. Male children were found to be more concurrently stunted and wasted (5.82%) compared to female children. WaSt cases are more prevalent among higher birth order children than lower birth order. Compared to mothers with some education, those without education are more likely to have WaSt children. Children who used unimproved toilet facilities and drank water from unimproved sources have a higher prevalence of WaSt than those who used improved toilet facilities and water sources. Similarly, children living in rural areas and belonging to socially disadvantageous populations have a higher prevalence of WaSt.

**TABLE 1:** Bivariate analysis of concurrent stunted and wasted (WaSt) children with their sociodemographic characteristics in India, 2019–21.

Variables	Weighted N)	Weighted (%)	WaSt (%)	*p*-value
Child characteristics				
Age of the child				<0.001
<6 months	17,767	9.06	2.37	
6–11	19,209	9.79	3.67	
12–23	38,744	19.75	6.48	
24–35	39,651	20.22	5.45	
36–47	39,825	20.31	5.85	
48–59	40,936	20.87	5.14	
Sex of the child				<0.001
Male	101,270	51.63	5.82	
Female	94,863	48.37	4.58	
Birth order				<0.001
1st	76,370	38.94	4.43	
2–4	109,682	55.92	5.57	
5 and above	10,081	5.14	7.30	
Size of the child at birth				<0.001
Large	36,673	18.89	4.85	
Average	137,252	70.68	4.87	
Small	20,259	10.43	8.09	
Had fever in the last 2 weeks				<0.001
No	169,442	86.44	5.08	
Yes	26,589	13.56	6.09	
Had a cough in the last 2 weeks				0.002
No	168,725	86.10	5.17	
Yes	27,235	13.90	5.55	
Had diarrhea in the last 2 weeks				<0.001
No	181,271	92.51	5.13	
Yes	14,681	7.49	6.41	
Maternal characteristics				
Maternal education				<0.001
No education	41,112	20.96	7.36	
Primary	24,114	12.29	6.39	
Secondary	100,440	51.21	4.77	
Higher	30,467	15.53	2.89	
Antenatal care				<0.001
None	8,920	6.00	6.90	
1–3	51,071	34.36	5.55	
4 or more	88,624	59.63	4.70	
Place of delivery				<0.001
Home	21,599	11.01	7.32	
Health facility	174,141	88.79	4.96	
Other	393	0.20	4.64	
Mother’s anemic level				<0.001
Severe	4,370	2.27	6.39	
Moderate	60,075	31.15	5.85	
Mild	51,277	26.59	5.24	
Not anemic	77,142	40.00	4.66	
Household characteristics				
Drinking water source				0.479
Unimproved	7,813	4.21	6.14	
Improved	177,960	95.79	5.21	
Toilet facility				<0.001
Unimproved	46,007	24.94	7.45	
Improved	138,439	75.06	4.52	
Household size				0.051
Small	903	0.46	5.57	
Medium	87,462	44.59	5.21	
Large	107,769	54.95	5.22	
Frequency of reading newspapers or magazines				<0.001
Not at all	139,987	71.37	5.78	
Less than once a week	35,333	18.01	3.99	
At least once a week	20,813	10.61	3.53	
Frequency of listening to the radio				<0.001
Not at all	173,198	88.31	5.30	
Less than once a week	16,572	8.45	4.78	
At least once a week	6,363	3.24	4.12	
Frequency of watching television				<0.001
Not at all	63,356	32.30	6.66	
Less than once a week	39,016	19.89	5.22	
At least once a week	93,761	47.80	4.24	
Cooking fuel				<0.001
Unclean	92,823	50.01	6.32	
Clean	92,790	49.99	4.17	
Wealth Index				<0.001
Poorest	47,729	24.33	7.82	
Poorer	42,827	21.84	5.78	
Middle	38,689	19.73	4.72	
Richer	36,473	18.60	3.67	
Richest	30,414	15.51	2.84	
Place of residence				<0.001
Urban	51,275	26.14	4.24	
Rural	144,858	73.86	5.57	
Religion				<0.001
Hindu	156,221	79.65	5.34	
Muslim	31,238	15.93	4.89	
Christian	4,224	2.15	4.39	
Others	4,450	2.27	3.97	
Social group				<0.001
Schedule caste	45,900	24.76	5.77	
Schedule tribe	19,414	10.47	7.31	
OBC	85,232	45.97	5.14	
None of them	34,842	18.79	3.63	

The p-values were obtained from the chi-square test.

The associations between the outcome (WaSt) and the explanatory variables are depicted in Table 2. The outcome of the model that incorporates all the explanatory factors chosen through gvselect (model III) indicates that female children have a 29% low risk of being a case of WaSt than male children (aOR = 0.71, 95% CI = 0.68, 0.74). When comparing children of different birth orders, those of the higher birth order are 1.2 times more likely to be WaSt cases (aOR = 1.20, 95% CI = 1.09, 1.33). Furthermore, compared to children who were larger at birth, smaller children have a 1.8-fold higher risk of becoming cases of WaSt (aOR = 1.81, 95% CI = 1.66, 1.97). Children who recently suffered from fever and diarrhea have significantly higher chances of being WaSt cases. Children of educated mothers have a lower chance of having WaSt, and this strongly correlates with the increased level of primary, secondary, and higher education of the mother; children of mothers with higher education have a 37% lower chance of being WaSt cases (aOR = 0.63, 95% CI = 0.57, 0.71). Children with access to improved toilet facilities have a 17% lower chance of being WaSt (aOR = 0.87, 95% CI = 0.82, 0.92). Furthermore, a strong correlation was observed between the wealth index and children’s WaSt instances, where children from wealthy families are 52% less likely to be WaSt cases (aOR = 0.48, 95% CI = 0.43, 0.55).

**TABLE 2:** Fixed and random effects result in the association of concurrently stunted and wasted children and stunting with their sociodemographic characteristics in India, 2019–21.

Variable	Model-0	Model-I aOR (95% CI)	Model-II aOR (95% CI)	Model III aOR (95% CI)
Child characteristics				
Age of the child				
<6 months		1 [1.00, 1.00]	1 [1.00, 1.00]	1 [1.00, 1.00]
6–11		1.40*** [1.24, 1.58]	1.49*** [1.30, 1.72]	1.46*** [1.27, 1.67]
12–23		2.62*** [2.36, 2.91]	2.91*** [2.58, 3.27]	2.78*** [2.47, 3.13]
24–35		2.07*** [1.86, 2.30]	2.34*** [2.07, 2.65]	2.16*** [1.92, 2.44]
36–47		2.17*** [1.95, 2.41]	2.41*** [2.13, 2.73]	2.25*** [2.00, 2.54]
48–59		1.90*** [1.71, 2.12]	2.07*** [1.82, 2.36]	1.98*** [1.75, 2.23]
Sex of the child				
Male		1 [1.00, 1.00]	1 [1.00, 1.00]	1 [1.00, 1.00]
Female		0.72*** [0.69, 0.75]	0.70*** [0.67, 0.74]	0.71*** [0.68, 0.74]
Birth order				
1st			1 [1.00, 1.00]	1 [1.00, 1.00]
2–4			1.09** [1.02, 1.16]	1.13*** [1.07, 1.19]
5 and above			1.11 [0.99, 1.24]	1.20*** [1.09, 1.33]
Size of the child at birth				
Large			1 [1.00, 1.00]	1 [1.00, 1.00]
Average			1.09* [1.02, 1.17]	1.07* [1.01, 1.14]
Small			1.85*** [1.68, 2.03]	1.81*** [1.66, 1.97]
Had fever in the last 2 weeks				
No			1 [1.00, 1.00]	1 [1.00, 1.00]
Yes			1.06 [0.97, 1.16]	1.13*** [1.05, 1.21]
Had a cough in the last 2 weeks				
No			1 [1.00, 1.00]	
Yes			1.03 [0.95, 1.13]	
Had diarrhea in the last 2 weeks				
No			1 [1.00, 1.00]	1 [1.00, 1.00]
Yes			1.14** [1.04, 1.25]	1.15** [1.06, 1.25]
Maternal characteristics				
Maternal education				
No education			1 [1.00, 1.00]	1 [1.00, 1.00]
Primary			0.95 [0.87, 1.04]	0.95 [0.88, 1.02]
Secondary			0.82*** [0.76, 0.89]	0.82*** [0.77, 0.88]
Higher			0.63*** [0.56, 0.72]	0.63*** [0.57, 0.71]
Antenatal care				
None			1 [1.00, 1.00]	
1–3			1.05 [0.94, 1.17]	
4 or more			1.07 [0.95, 1.19]	
Place of delivery				
Home			1 [1.00, 1.00]	
Health facility			0.90* [0.83, 0.98]	
Others			1.08 [0.63, 1.87]	
Mother anemic level				
Severe			1 [1.00, 1.00]	1 [1.00, 1.00]
Moderate			0.95 [0.80, 1.12]	1.01 [0.87, 1.18]
Mild			0.93 [0.79, 1.10]	0.97 [0.83, 1.13]
Not anemic			0.86 [0.73, 1.02]	0.9 [0.77, 1.04]
Household characteristics				
Drinking water source				
Unimproved				1 [1.00, 1.00]
Improved				1.11* [1.00, 1.22]
Toilet facility				
Unimproved			1 [1.00, 1.00]	1 [1.00, 1.00]
Improved			0.85*** [0.79, 0.90]	0.87*** [0.82, 0.92]
Household size				
Large			1 [1.00, 1.00]	1 [1.00, 1.00]
Medium			1.34 [0.93, 1.94]	1.34 [0.93, 1.93]
Small			1.45* [1.00, 2.10]	1.42 [0.99, 2.05]
Frequency of reading newspapers or magazines				
Not at all			1 [1.00, 1.00]	
Less than once a week			0.96 [0.89, 1.05]	
At least once a week			1.04 [0.92, 1.16]	
Frequency of listening to the radio				
Not at all			1 [1.00, 1.00]	
Less than once a week			0.96 [0.87, 1.07]	
At least once a week			0.92 [0.78, 1.08]	
Frequency of watching television				
Not at all			1 [1.00, 1.00]	1 [1.00, 1.00]
Less than once a week			1.01 [0.94, 1.09]	1.01 [0.95, 1.08]
At least once a week			0.95 [0.88, 1.02]	0.94 [0.88, 1.00]
Cooking fuel				
Unclean			1 [1.00, 1.00]	
Clean			1.01 [0.94, 1.08]	
Wealth Index				
Poorest			1 [1.00, 1.00]	1 [1.00, 1.00]
Poorer			0.81*** [0.75, 0.87]	0.82*** [0.77, 0.88]
Middle			0.70*** [0.63, 0.77]	0.72*** [0.66, 0.78]
Richer			0.53*** [0.47, 0.60]	0.55*** [0.50, 0.61]
Richest			0.46*** [0.39, 0.53]	0.48*** [0.43, 0.55]
Place of residence				
Urban			1 [1.00, 1.00]	1 [1.00, 1.00]
Rural			0.91* [0.84, 0.99]	0.89** [0.83, 0.96]
Religion				
Hindu			1 [1.00, 1.00]	1 [1.00, 1.00]
Muslim			1.01 [0.92, 1.11]	0.98 [0.90, 1.06]
Christian			0.54*** [0.48, 0.62]	0.53*** [0.47, 0.61]
Other			0.67*** [0.57, 0.79]	0.64*** [0.55, 0.75]
Social group				
Schedule caste			1 [1.00, 1.00]	1 [1.00, 1.00]
Schedule tribe			1.14** [1.05, 1.24]	1.11** [1.03, 1.20]
OBC			1.02 [0.95, 1.10]	1 [0.94, 1.07]
None of them			0.79*** [0.72, 0.87]	0.77*** [0.71, 0.84]
Random effect model				
PSU variance (95% CI)	0.63	0.64	0.43	0.55
ICC	0.16	0.16	0.12	0.14
Wald chi-square	Reference	673.04	1881.46	2131.98
Model fitness				
Log-likelihood	−38309.93	−37944.86	−24350.15	−32342.34
AIC	76623.87	75905.72	48792.3	64758.69

*p < 0.05, **p < 0.01, ***p < 0.001.

## Discussion

In this multilevel analysis, we estimated the concurrent occurrence of WaSt among children under 5 years of age and its association with demographic characteristics in the Indian context. The results provide insight into the role of various socioeconomic factors in the development of these conditions and may be informative for the development of effective interventions to reduce WaSt. The prevalence of WaSt among children under five in India was 5.2%, and all children with WaSt were also underweight in the current analysis. Khara et al. reported a pooled prevalence rate of 3%, where they included data from 84 countries [19]. There was a decrease in the current estimates of WaSt (5.6%) compared to 2005–06 (8.7%) in India. The data from Brazil also show similar declining trends in WaSt rates [20]. Strategies such as nutrition intervention programs and integrated management of childhood illnesses might have caused this improved trend in WaSt prevalence. In the current analysis, the highest WaSt prevalence rate of 8% was found at approximately 19 months of age, with an earlier peak among boys (17 months) than among girls (19 months). A higher age (12–23 months aOR = 2.78) has been shown to be associated with a greater risk of WaSt in the present study, which is consistent with the findings of Khaliq et al. [21] A pooled analysis of previous studies reported a greater risk of WaSt among the children in 12–36 months of age [19]. However, Saaka et al. from Ghana reported findings similar to those in the present study, in which they found a strong association between WaSt in children of 12–23 months of age and with children aged 0–5 months [22].

Female children were found to have a 29% lower prevalence of WaSt than male children in the present study. Most studies from the past have shown that boys had the worst undernutrition indices: wasting, stunting, underweight [23], and WaSt [2, 19] when compared to girls. Although there is a significant history of gender bias against women in India, included food allocation at the household level [24], the lower prevalence of WaSt may be due to the effectiveness of government interventions to alleviate bias against girls or may be due to the inherent increased survival capacity of the female children compared to male children in India. An inquiry about the preference towards male child in the surveyed population could have brought about a better understanding of the reasons behind this finding. The finding gained importance in light of the absence of a truly gender-equitable approach in nutrition programs, as boys seem to be more vulnerable to undernutrition. Although inequities in nutrition intake by women have been documented, the possible reasons for the male child’s vulnerability to undernutrition remain to be explored [25]. Nutrition policies and programs targeting under-five-year-old children will benefit from this information on gender-specific differences in childhood growth and nutrition. Another possible reason could be a higher exposure of male children to outdoor environments when compared to female children, where they are potentially more exposed to the microbial infections such as soil transmitted helminths (STH) known to cause growth deficits and stunting; however, this needs to be explored with specific inquiries in future surveys. In the current analysis, while boys had a relatively higher proportion of mild and moderately severe WaSt than girls in the age group below 30 months, girls had a higher rate of severe WaSt than boys between 30 and 59 months. The reason for such differential findings of WaSt prevalence and severity between sexes and age groups should be studied in the future by intersectional analysis.

Birth weight has been implicated as a significant factor in the nutritional status of children under 5 years of age. Although the average and smaller babies at birth were found to have a significantly higher rate of WaSt in the current analysis, Islam et al. from Bangladesh, in contrast, reported that average or large babies were at higher odds of undernutrition [26]. In addition to birth weight, the order of birth along with birth interval can also affect the nutritional status of children. A higher order of birth was found to have a significant association with the prevalence of WaSt in India, which is similar to a finding reported from Bangladesh [26]. It has been suggested that a higher order of birth, in combination with food insecurity, may lead to significant undernourishment in children [27, 28].

In India, there are multiple castes that are broadly grouped under Scheduled Castes (SC), Scheduled Tribes (ST), Other Backward Classes (OBC), and others. While SC/ST fared worse than the OBC and others in 2015–16 [14], the current data suggests that SC/ST along with OBCs suffered worse outcomes than the others. Caste is an important determinant of health, socioeconomic and political wellbeing in India and is being followed in the Indian subcontinent since ancient times. It is “the system of dividing people in a society into different social classes” [29] and is determined on the basis of birth in a particular family. In this social hierarchical system, the populace is grouped into the so-called “lower” and “upper” castes. It has been shown that people from the so-called lower castes consume a lesser quantity of fruits and vegetables than their counterparts [30]. Although the under-nutritional aspects of SC/ST have been brought out [31, 32], the poor nutritional status in the OBC population found in the present study and the determinants need further evaluation.

The incidence of fever and diarrhea in the last 2 weeks, maternal education below secondary level, inadequate toilet facilities, large household size, lower quintiles of wealth index, residing in an urban area, and followers of Hindu/Muslim religions were significantly associated with the higher prevalence of WaSt in India. Urban background, poverty, no education, and higher birth order were also reported as significant predictors from 2015 to 2016 in India [14]. Studies from Bangladesh and Ethiopia reported a significant impact of childhood infections on CFM [26, 33]. In contrast to our findings, the mother’s education was not reported as a significant factor in stunted children from Bangladesh and Pakistan [21, 26]. The higher education status of the mother determines the health and nutrition behavior toward herself and the child. A lower socioeconomic status was a significant determinant of WaSt from other countries as well [21, 26]. Better income levels and socioeconomic status enables better access to safe water, healthy food, and improved sanitation practices. Thus, in a broad sense, socioeconomic factors also impact the other determinants of undernutrition. Khan et al. reported no significant association between sanitation and undernourished children with CFM [14], unlike our findings. Safe drinking water has been found to protect against WaSt [33]. Lastly, religion has also been shown to have a significant association with undernutrition according to the earlier rounds of the NFHS [32].

The strengths of the present analysis are that it is based on the analysis of a large dataset representative of India collected following a robust sample size, sampling technique, and adherence to standardized anthropometric and data collection methods. Multiple models were used to improve robustness to identify the independent and significant predictors of WaSt. However, the analysis is not devoid of limitations. First, considering the secondary nature of the data used, the more direct predictors such as the nutritional intake could not be assessed. A cross-sectional design of the data collection limits the temporal linkage between WaSt and the predictors such as education, wealth, residential status, and household size. Owing to the interview-based collection of data, with or without verification of the records, the biases of recall and social desirability cannot be ruled out. Lastly, India is a diverse country, and the WaSt rates and their predictors which may vary due to differences in the health pattern and profile of each state of India could not be brought out in the current analysis.

### Conclusion

In general, children under five in India had a concurrent prevalence of wasting and stunting of 5.2%, with a declining trend from the past. Male children had a negative association with WaSt. Social factors such as caste (SC/ST/OBC), religion (Hindus and Muslims), maternal education levels, poor socio-economic status of the family, and poor sanitation were found to be significant predictors of WaSt in children under 5 years of age in India. National programs and customized strategies targeting these factors and social groups must be implemented to improve the nutritional status of children under 5 years of age in India. Furthermore, analytical and primary research must be conducted to evaluate the root cause and factors related to inequity against male children in undernutrition and the relationship between age, sex, and severity in determining WaSt prevalence in Indian settings.

## Data Availability

Data are available at the Demographic Health Survey (DHS) data repository through https://dhsprogram.com and could be accessed upon a data request subject to non-profit and academic interest only.
